# Prenatal exposure to acetaminophen and adolescent assessment of behavior: Discrepancies by age and reporter

**DOI:** 10.3389/fphar.2023.1084781

**Published:** 2023-03-02

**Authors:** Rashida S. Smith-Webb, Ruby Barnard-Mayers, Martha M. Werler, Samantha E. Parker

**Affiliations:** Department of Epidemiology, Boston University School of Public Health, Boston, MA, United States

**Keywords:** acetaminophen, prenatal, behavior, externalizing, internalizing

## Abstract

Acetaminophen, which is one of the most commonly used medications during pregnancy, has been linked to adverse neurodevelopmental outcomes among offspring during childhood. Less is known about associations with outcomes occurring later in adolescence.

**Methods:** We conducted a follow-up study of children born between 1996 and 2002. Data on illnesses and medications, including acetaminophen, during pregnancy were collected through a standardized interview after delivery. Behavioral assessments were conducted at two subsequent time points, childhood (ages 5–10) and adolescence (ages 11–17). Outcomes examined included internalizing, externalizing, and total behavior problems based on the parent-completed Child Behavior Checklist (CBCL), the teacher-completed Teacher Report Form (TRF), and the youth-completed Youth Self Report (YSR, adolescent follow-up only). Adjusted linear regression models were used to calculate mean differences (MD) and 95% confidence intervals (95% CI) in T-scores comparing those with prenatal acetaminophen exposure to those without. Stabilized inverse probability weights were used to account for attrition.

**Results:** Among the 216 mother-child dyads with completed parent and teacher behavioral assessments at both childhood and adolescence, prenatal acetaminophen exposure was not associated with behavioral problems according to either parent or teacher assessments. Modest increases in externalizing and total behavior problems were observed according to youth report (MD: 1.9). Compared to associations observed during the childhood follow-up, associations at adolescence were attenuated according to parent-report.

**Conclusion:** Reported associations between prenatal acetaminophen exposure and behavioral outcomes were not consistent over time nor between reporters.

## Introduction

Acetaminophen has long been considered the safest pain reliever to take during pregnancy (Society for Maternal-Fetal Medicine Publications Committee, 2017), with an estimated 60% of pregnant patients reporting use at some point during pregnancy (Bandoli et al., 2019). However, recent studies have suggested that prenatal acetaminophen use may be a risk factor for adverse neurodevelopmental outcomes among offspring, including attention deficit hyperactivity disorder (ADHD), autism, behavioral problems, and executive function (Brandlistuen et al., 2013; Liew et al., 2014; Thompson et al., 2014; Liew et al., 2015; Andrade, 2016; Liew et al., 2016; Stergiakouli et al., 2016; Ystrom et al., 2017; Rifas-Shiman et al., 2020). Studies of prenatal exposures and childhood neurodevelopment are often fraught with limitations, including unmeasured confounding, challenges with outcome assessment, and exposure misclassification; as such, the validity of these studies has been called into question (Damkier et al., 2015; Damkier, 2020).

In contrast to clinically diagnosed outcomes, subclinical measures of childhood behavior are assessed with a variety of instruments and reporters, without a consistent case definition. A limitation of the existing literature on subclinical outcomes is the reliance on a single reporter of behavior, most frequently the parent or caregiver. While parental assessments of behavior provide valuable information, child functioning varies from one environment to another, due in part to different relationships and interactions (Achenbach et al., 1987). Thus, assessments of the child in different settings, such as home and school, provide a broader picture of child’s behavior where inconsistencies are common. Assessments with parent or caregiver as the reporter generally produce poorer scores of child’s behavior than those from a teacher across numerous instruments (Youngstrom et al., 2000; Stone et al., 2013; Falt et al., 2018), and parental stress (Youngstrom et al., 2000) and socioeconomic indicators (Stone et al., 2013) have been repeatedly identified sources of discrepancies between reporters. The magnitude of such discrepancies also differs by child age (Achenbach et al., 1987; Murray et al., 2018).

The potential impact of prenatal exposure to acetaminophen on behavioral outcomes has largely been studied among elementary-school aged and early adolescent children. Studies that have explored these associations in older adolescents report potentially waning associations over time (Tovo-Rodrigues et al., 2018; Golding et al., 2019), but explanations for attenuation of an effect with increasing age are lacking. Additional data are needed to further explore both repeated measurements during childhood and adolescence with multiple reporters.

To address the limitations (i.e., single reporter, dependent error, selection bias), we conducted a follow-up study with repeated measurements collected during childhood and adolescent by three reporters. The objective of this study was to investigate the association between prenatal acetaminophen exposure and behavioral problems during childhood and at adolescence, assessed by a parent, a teacher, and the child themselves.

## Materials and methods

### Data

**FIGURE 1:**
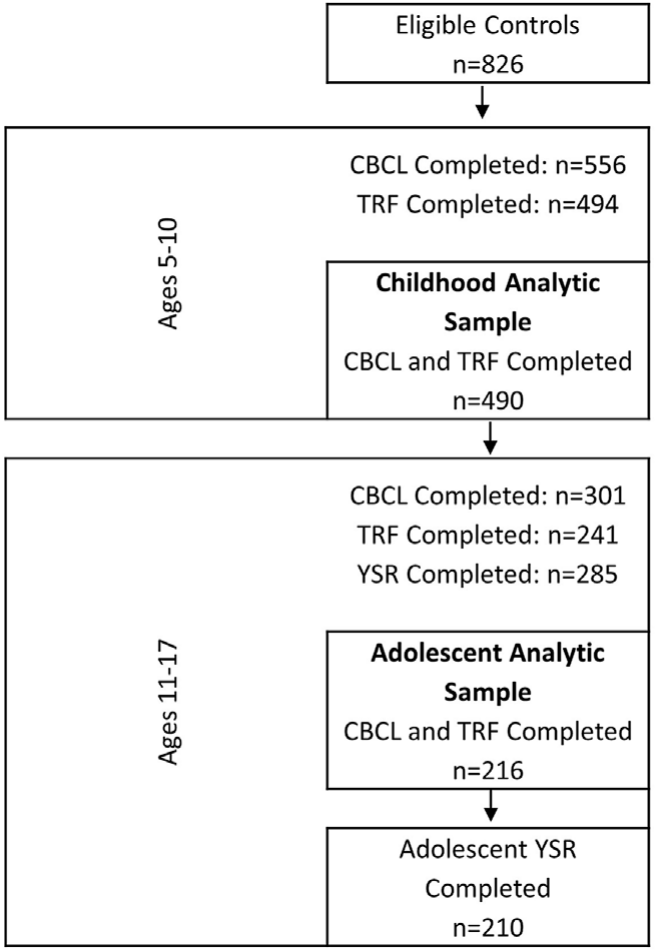
Flow diagram of eligible controls from the case-control study and number of participants at childhood and adolescent behavioral assessments.

The data used in this analysis come from a case-control study of prenatal exposures and craniofacial malformation in infants born between 1996 and 2002. Details of the study design have been described previously (Werler et al., 2004). Briefly, cases of hemifacial microsomia were ascertained from 26 craniofacial centers across the United States and Canada. Controls were live-born infants without a major malformation, randomly selected from the same birth population as cases and matched on birth year, pediatric practice, or practices within the same zip code. Information on maternal demographics, reproductive history, illnesses during pregnancy, and medication use was collected through an interview conducted within 4 years of delivery. Participants of the original study were re-contacted to participate in a follow-up study that collected information on neurodevelopment during childhood (5–10 years) and adolescence (11–17 years). The analytic cohort for this analysis includes the 216 singleton control children with data on maternal exposures collected on average 12 months after delivery (IQR: 3–18 months) and neurodevelopment assessments in both childhood and adolescence (Figure 1). This study was approved by the Institutional Review Board (IRB) at Boston University.

### Exposure

Information on acetaminophen use during pregnancy was collected *via* standardized telephone interview after delivery and prior to conduct of any neurodevelopmental assessments of the child. Mothers were asked about medication use for upper respiratory infection (cold, flu, and bronchitis), pain, fever, headaches (migraine and sinus), and other selected indicators for the 6-month period beginning the 1 month prior to their last menstrual period through the fifth month of pregnancy. Mothers were also asked if they have used any of a list of specific medications, including Tylenol and acetaminophen. For each medication reported, information was reported on the product name, start and stop dates, and frequency and duration. When available, confirmation of the exact medication formulation was done through product packaging or identification from medication booklet used to aid in recall. The Slone Drug Dictionary was used to classify all medications reported (Kelley et al., 2003). Mothers were considered exposed to acetaminophen if they reported any medication that contained acetaminophen alone or in combination with other medications during pregnancy. Acetaminophen use reported prior to the last menstrual period was not included. Maternal acetaminophen use served as a proxy for prenatal exposure and was categorized as no exposure (reference) or any exposure, and among any exposure, chronic exposure was defined as >28 days during pregnancy.

### Outcome

The primary outcome of interest for this study was report of behavioral problems during adolescence. Adolescent behavior was assessed using the school-age Achenbach System of Empirically Based Assessments (ASEBA), which allows for comparison of reports across reporters or informants (Achenbach and Rescorla, 2001). The assessments used include the parent-completed Child Behavior Checklist (CBCL), the teacher-completed Teacher Report Form (TRF), and the self-completed Youth Self Report (YSR). The forms cover a range of behavioral components for which responses are tallied to provide measures for the broadband scales, including internalizing behavioral problems (consisting of anxious/depressed signs, withdrawn/depressed signs, and somatic complaints), externalizing behavioral problems (consisting of rule-breaking and aggressive behavior) and total behavior problems. In this analysis, we focus on the three composite broadband scales. The school-age CBCL and TRF assessments were also completed during the childhood follow-up of this cohort. T-scores, which are normed to gender (Achenback and Rescorla, 2001), for each assessment were calculated and serve as a measure of the degree to which an individual deviates from a normative sample (mean = 50, SD = 10) with higher scores indicative of worse behavior. We also dichotomized the broadband scale T-scores to reflect scores in the borderline clinical and clinical range (T ≥ 60). Children with scores from 60 to 63 are considered to be in the borderline clinical range, with scores above 64 indicating a child within the clinical range.

### Analysis

We examined the distribution of maternal characteristics in the analytic sample overall and by acetaminophen use during pregnancy. For assessments collected among adolescents, we calculated mean scores for internalizing, externalizing and total (broadband) scales of the CBCL, TRF, and YSR, and Pearson correlation coefficients of scores between pairs of reporters (parent-teacher, parent-youth, teacher-youth). We used linear regression models to calculate the unadjusted and adjusted mean differences (MD) and 95% confidence intervals (CIs) for the broadband scales at adolescence according to the three reporters (parent, teacher, and youth). Mothers reporting no acetaminophen use during pregnancy was the reference group. We identified confounders for adjustment *a priori* based on prior literature, which included maternal age, education, marital status, parity, drinking, and smoking.

To address concerns about cohort attrition leading to selection bias, we conducted weighted analyses using stabilized inverse-probability weighting (SIPW) based on the probability of participation and compared these weighted results to the adjusted regression results. Variables used to calculate the weights using a logistic regression model were maternal age and paternal age at delivery, infant sex, maternal race, marital status, maternal education, alcohol drinking and smoking during pregnancy, parity, gravidity, acetaminophen exposure, headache, and planned pregnancy. The c-statistic of the model was 0.696. We then divided the predicted probability of participation by the proportion of mothers that participated to create stabilized weights, which can avoid the influence of extreme weights.

For the dichotomized broadband scales using deviant cut points (T-scores ≥60), we conducted log-binomial regression models with a Poisson distribution to calculate unadjusted and stabilized inverse probability weighted risk ratios (RR). We repeated the analysis for CBCL and TRF T-scores collected at the childhood assessment in order to compare results between the childhood and adolescent time frames.

We also conducted sensitivity analyses to examine the impact of adjustment for indication (headache, upper respiratory infection, and pain) and also examined associations between chronic use of acetaminophen during pregnancy (>28 days) and behavioral outcomes. To test the robustness of our findings with regards to inclusion criteria, we reexamined associations between prenatal acetaminophen exposure and behavioral outcomes among respondents who had at least one behavioral assessment completed in adolescence. We used the previously calculated weights for the cohort completing both assessment at childhood and adolescence.

## Results

There were 826 eligible controls of which 216 had both parent and teacher assessments completed during childhood and adolescence. Mothers included in the analytic sample were predominantly white (80.1%), with at least 16 years of education (56.0%), and married (97.1%) (Table 1). Among these women, 66.2% reported any acetaminophen use during pregnancy. Acetaminophen users were more likely to be non-Hispanic white (86.0% vs. 68.5%) and have at least 16 years of education (58.0% vs. 52.1%) but less likely to report smoking (11.9% vs. 19.2%) and drinking (32.9% vs. 35.6%) during pregnancy compared to non-users (Table 1).

**TABLE 1:** Characteristics of eligible controls, participants at childhood and adolescent behavioral assessment, and by acetaminophen use during pregnancy.

	Eligible controls *n* = 826			Total *n* = 216			Acetaminophen users *n* = 143			Acetaminophen Non-users *n* = 73	
Race/Ethnicity	n	%		n	%		n	%		n	%
White, non-hispanic	540	65.4		173	80.1		123	86.0		50	68.5
Black, non-hispanic	161	19.5		22	10.2		7	4.9		15	20.6
Hispanic	87	10.5		14	6.5		9	6.3		5	6.9
Other	38	4.6		7	3.2		4	2.8		3	4.1
Age at conception											
≤25	260	31.5		34	15.7		20	14.0		14	19.2
26–34	441	53.4		144	66.7		99	69.2		45	61.6
≥35	125	15.1		38	17.6		24	16.8		14	19.2
Education											
≤12 years	323	39.1		47	21.8		25	17.5		22	30.1
13–15 years	186	22.5		48	22.2		35	24.5		13	17.8
≥16 years	316	38.3		121	56.0		83	58.0		38	52.1
Marital status											
Married	722	87.4		198	91.7		131	91.6		67	91.8
Single/Divorced	104	12.6		18	8.3		12	8.4		6	8.2
Body mass index											
<18.5	30	3.6		6	2.8		4	2.8		2	2.7
18.5–24.9	509	61.6		135	62.5		94	65.7		41	56.2
25–29.9	165	20		40	18.5		22	15.4		18	24.7
≥30	98	11.9		34	15.7		23	16.1		11	15.1
Missing	24	2.9		1	0.5		0	0.0		1	1.4
Alcohol drinking	241	29.2		73	33.8		47	32.9		26	35.6
Missing				1	0.5		1	0.7		0	0.0
Smoking	114	13.8		31	14.4		17	11.9		14	19.2
Parity											
0	358	43.3		92	42.6		58	40.6		34	46.6
1	291	35.2		82	38.0		56	39.2		26	35.6
2+	177	21.4		42	19.4		29	20.3		13	17.8
Infant sex											
Male	423	51.2		101	46.7		66	46.2		35	48.0
Female	403	48.8		115	53.2		77	53.9		38	52.1
Indication											
Headache	460	55.7		132	61.1		110	76.9		22	30.1
Upper respiratory	474	57.4		137	63.4		100	69.9		37	50.7
Pain	112	13.6		36	16.7		26	18.2		10	13.7
Acetaminophen use											
Any	487	59.0		143	66.2		—	—		—	—
None	339	41.0		73	33.8		—	—		—	—

Table 2 shows the mean scores for each of the three broadband scales during adolescence according to the three reporters (parent, teacher, and youth). Parent report (CBCL) had the highest mean scores for internalizing behaviors, while the teacher report (TRF) had the highest mean scores for externalizing behaviors. Parent and youth (YSR) scores were more strongly correlated (r: 0.38–0.40) than parent and teacher scores (r: 0.20–0.24).

**TABLE 2:** Mean T-Scores and pearson correlation coefficients for CBCL, TRF, and YSR assessments collected during adolescents only.

	Analytic sample *n* = 216		
	T-score	Correlation coefficient	
	Mean (Range)	With CBCL	With TRF
Internalizing			
CBCL	49.3 (33–78)	----	----
TRF	48.3 (37–73)	0.20	----
YSR	48.9 (27–74)	0.40	0.21
Externalizing			
CBCL	46.2 (33–74)	----	----
TRF	48.4 (41–83)	0.22	----
YSR	46.1 (29–72)	0.38	0.29
Total problems			
CBCL	47.0 (24–75)	----	----
TRF	48.3 (32–77)	0.24	----
YSR	48.1 (26–73)	0.38	0.28

**FIGURE 2:**
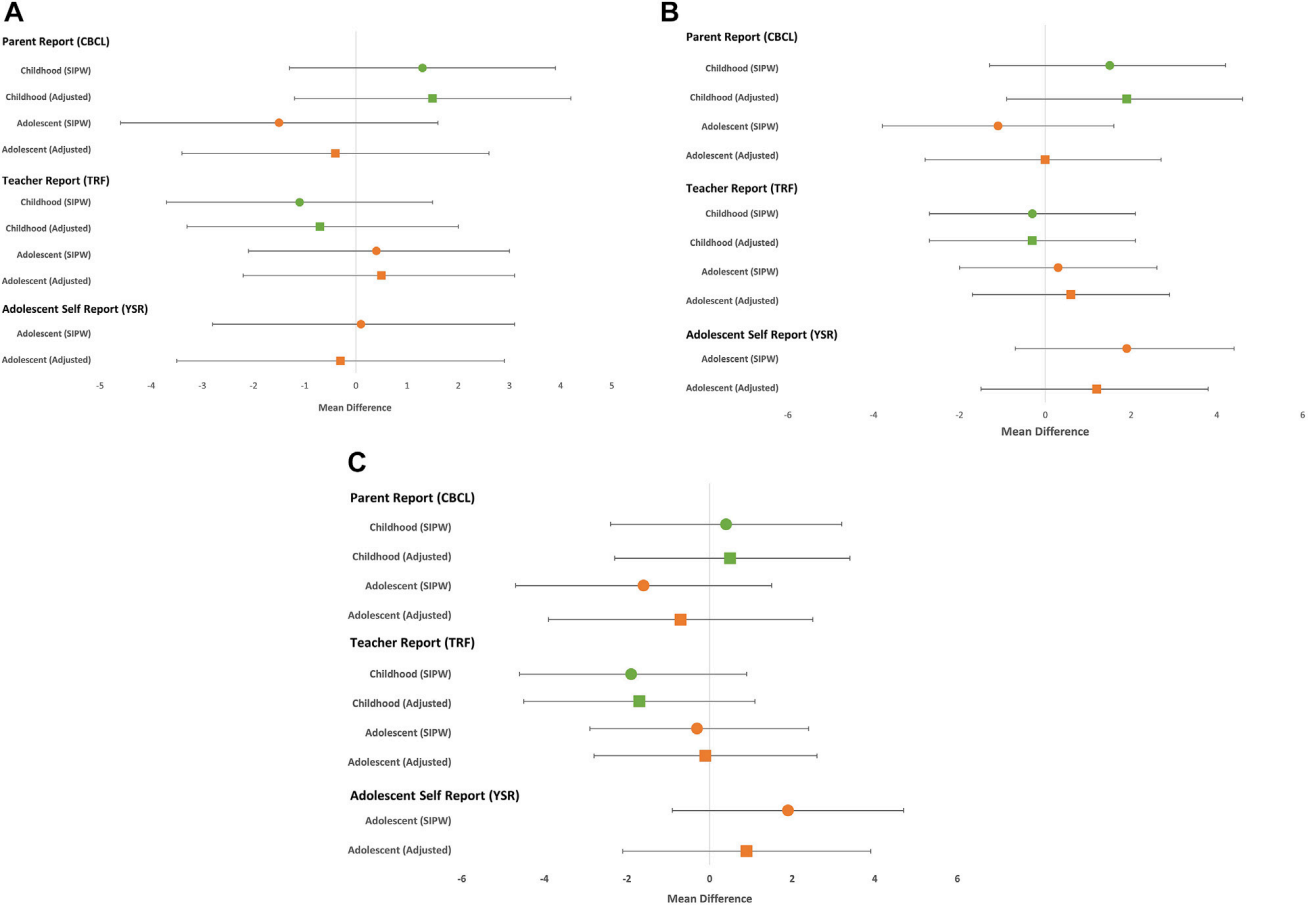
Mean differences and 95% confidence for internalizing **(A)** externalizing **(B)** and total **(C)** behavior problems comparing prenatal exposure to acetaminophen to no prenatal exposure.

Adjusted mean differences for all CBCL and TRF reported broadband scales were generally null (Figure 2). According to youth self-report during adolescence, mean scores for externalizing and total behavior were higher among those with prenatal acetaminophen exposure (YSR Adjusted MD: 1.2 and 0.9, respectively). Models weighted to account for differences in participation resulted in decreases in the mean differences on the CBCL and TRF scales and increases in the YSR scales. In comparison to the childhood assessments, the mean differences for prenatal acetaminophen exposure and adolescent behavior decreased and even reversed direction according the CBCL. According to the TRF, there was less variability in the observed mean differences between childhood and adolescence with no increase in behavioral problems associated with prenatal exposure to acetaminophen observed at any time point (Figure 2).

Using a dichotomous outcome to identify scores in the borderline and clinical range, a similar pattern between childhood and adolescence was observed, with associations attenuating from childhood to adolescence on the CBCL (Total Problems RR: 1.8 and 1.0, respectively) and associations slightly increasing from unadjusted to weighted analyses on the YSR at adolescence (Total Problems RR: 1.0 and 1.5, respectively). The pattern for TRF was less clear, with the risk ratio for internalizing behavioral problems increasing between childhood and adolescence (RR: 0.8 and 1.5, respectively) and no major change in externalizing and total broadband scales. Of note, the proportion of our sample meeting the borderline/clinical threshold increased across all broadband scales from childhood to adolescence, but remained small, thereby leading to imprecise estimates (Table 3).

**TABLE 3:** Risk ratios and 95% confidence intervals for prenatal exposure to acetaminophen versus no exposure among children and adolescents within the borderline/clinical range for broadband scores by parent and teacher report at childhood and adolescence and youth report at adolescence.

	n (%)^a^	Unadjusted RR (95% CI)	Adjusted + SIPW^b^ RR (95% CI)
CBCL (*n* = 216)			
Internalizing			
Childhood	21 (9.7)	1.6 (0.6, 4.5)	0.9 (0.4, 2.1)
Adolescent	34 (15.7)	0.9 (0.5, 1.9)	0.6 (0.3, 1.0)
Externalizing			
Childhood	20 (9.3)	2.0 (0.7, 6.1)	1.2 (0.5, 3.0)
Adolescent	22 (10.2)	1.1 (0.4, 2.7)	0.6 (0.3, 1.3)
Total problems			
Childhood	18 (8.3)	2.6 (0.7, 8.8)	1.8 (0.6, 5.9)
Adolescent	28 (13.0)	1.5 (0.7, 3.6)	1.0 (0.5, 2.1)
TRF (*n* = 216)			
Internalizing			
Childhood	27 (12.5)	1.0 (0.5, 2.3)	0.8 (0.4, 1.7)
Adolescent	32 (14.8)	1.5 (0.7, 3.4)	1.5 (0.7, 3.2)
Externalizing			
Childhood	23 (10.7)	1.0 (0.4, 2.3)	0.9 (0.4, 2.1)
Adolescent	25 (11.6)	0.9 (0.4, 2.1)	1.1 (0.5, 2.2)
Total problems			
Childhood	30 (13.9)	0.8 (0.4, 1.6)	0.8 (0.4, 1.7)
Adolescent	26 (12.0)	0.7 (0.3, 1.5)	0.5 (0.2, 1.1)
YSR (*n* = 210) adolescence only			
Internalizing	41 (19.5)	1.0 (0.5, 2.0)	1.5 (0.7, 3.1)
Externalizing	19 (9.1)	1.2 (0.4, 3.0)	1.8 (0.6, 5.5)
Total problems	34 (16.2)	1.0 (0.5, 2.0)	1.5 (0.7, 3.2)

^a^ Number and percent of participants in the borderline/clinical range.

^b^ SIPW (stabilized inverse probability weights) is weighted and adjusted for age, education, marital status, drinking, smoking, parity.

We also conducted sensitivity analyses to examine the impact of adjustment for indication on the results, as well as examining chronic use (>28 days) as the exposure. Adjustment for indication resulted in the mean differences moving in a downward trend according to all reporters; evidence of positive confounding by indication. Compared to the primary analysis, the results for chronic use showed an increase in associations between prenatal acetaminophen exposure and externalizing behavioral problems according to both parent and teacher report. (CBCL: 0.6 (−3.4, 4.5) and TRF 1.2 (−2.2, 4.7) (Table 4). Confidence intervals from these analyses were wide indicating imprecise estimates. Table 4 also shows the results of the sensitivity analysis which was conducted among a larger sample of participants with at least one available adolescent behavior assessment. Associations for teacher and youth report were similar to the primary analysis, while associations with parent-reported outcomes were stronger.

**TABLE 4:** Mean differences and 95% confidence intervals for broadband T-Scores comparing prenatal exposure to acetaminophen to No prenatal exposure, sensitivity analyses.

Original sample					Sample with ≥1 adolescent measure	
	Primary analysis^a^	Indication adjusted^b^	Chronic use^c^			Adjusted + SIPW^a^
CBCL (*n* = 216)					CBCL (*n* = 301)	
Internalizing	−1.5 (−4.6, 1.6)	−2.6 (−6.1, 1.00)	−1.0 (−5.6, 3.6)		Internalizing	1.2 (−1.3, 3.7)
Externalizing	−1.1 (−3.8, 1.6)	−1.4 (−4.5, 1.7)	0.6 (−3.4, 4.5)		Externalizing	0.7 (−1.6, 2.9)
Total problems	−1.6 (−4.7, 1.5)	−2.9 (−6.6, 0.7)	−1.8 (−6.4, 2.9)		Total problems	0.9 (−1.7, 3.6)
TRF (*n* = 216)					TRF (*n* = 241)	
Internalizing	0.4 (−2.1, 3.0)	0.3 (−2.7, 3.3)	0.2 (−3.6, 4.0)		Internalizing	0.4 (−2.0, 2.8)
Externalizing	0.3 (−2.0, 2.6)	−0.1 (−2.8, 2.6)	1.2 (−2.2, 4.7)		Externalizing	0.2 (−1.9, 2.3)
Total problems	−0.3 (−2.9, 2.4)	−0.9 (−3.9, 2.1)	0.5 (−3.4, 4.4)		Total problems	−0.4 (−2.9, 2.1)
YSR (*n* = 210)					YSR (*n* = 285)	
Internalizing	0.1 (−2.8, 3.1)	−1.3 (−4.7, 2.2)	−0.7 (−5.2, 3.8)		Internalizing	1.0 (−1.5, 3.5)
Externalizing	1.9 (−0.7, 4.4)	1.0 (−2.0, 3.9)	−0.1 (−3.8, 3.7)		Externalizing	2.0 (−0.2, 4.2)
Total problems	1.9 (−0.9, 4.7)	0.1 (−3.2, 3.3)	−0.2 (−4.5, 4.0)		Total problems	2.1 (−0.3, 4.5)

^a^ SIPW (stabilized inverse probability weights) is weighted and adjusted for age, education, marital status, drinking, smoking, parity.

^b^ Additionally adjusted for headache, upper respiratory infection, and pain.

^c^ Chronic use defined as ≥28 days during pregnancy.

## Discussion

The existing literature on neurodevelopmental outcomes in relation to prenatal acetaminophen exposure has overwhelmingly focused on behavior in early childhood, with many of these studies reporting increases in adverse neurocognitive outcomes. However, there is sparse literature examining associations in adolescence. Accordingly, it is important to understand the long-term safety profile to help inform clinician and patient decision-making.

In this study of prenatal acetaminophen exposure and behavioral outcomes during childhood and adolescence, we found that while associations were slightly stronger based on the parent-completed CBCL compared to the teacher-completed TRF during childhood, these estimates were mostly attenuated during adolescence. Our findings are consistent with previous studies, which also did not find associations between prenatal acetaminophen exposure and neurocognitive outcomes beyond the age of 7, (Bauer et al., 2018; Golding et al., 2019). Similarly, other studies have reported a decrease in ADHD-related symptoms between 6, 7, and 11 years of age (Thompson et al., 2014; Tovo-Rodrigues et al., 2018).

To account for selection bias due to loss to follow-up and non-participation, we used weighting. Associations were generally attenuated or decreased after weighting, indicating that failure to account for selection bias due to lack of participation resulted in larger associational measures, particularly for parent- and self-reported outcomes. Weighted estimates for teacher-reported outcomes were virtually unchanged from unweighted estimates. These findings suggest that parents/caregivers who are more likely to participate in the study report more behavioral problems in their children on average.

The discrepancy in parent- and teacher-reported behavioral outcomes, might be due in part to the observed environment (Hartman et al., 2007). Certainly, children’s behaviors at home may differ from at school, which can contribute to inconsistencies in reporting. In addition, parent and teacher relationships with the child and the duration of interactions are likely to vary substantially, from one context to another (Achenbach et al., 1987). These observed differences further demonstrate the importance of collecting multi-informant assessments to not only develop a more complete picture of the nature, extent, and severity of the behavioral problem, but to limit producing biased estimates.

We previously published data from this cohort on the childhood outcomes, in which we also observed stronger associations on CBCL than TRF (Parker et al., 2020). In that analysis, we used a larger sample including all children with at least one behavioral assessment during childhood. Similar to the present analysis, restriction to participants with both CBCL and TRF assessments resulted in null associations. Both analyses also showed a larger decrease in mean differences upon adjustment for indication. In both the prior and present analysis, our findings, particularly those according to parent-report, were sensitive to sample selection and weighting, further supporting a selection bias and also underscoring the need to use multi-informant assessments.

In contrast to parent and teacher reports, self-reported behavior problems were more strongly associated with prenatal acetaminophen exposure after accounting for attrition. Interestingly, slight increases in self-reported YSR externalizing and total behavioral mean scores were observed among those with prenatal exposure to acetaminophen. Our findings are consistent with the only other known study including self-reported behavior that similarly reported slightly higher associations between prenatal acetaminophen exposure and Strengths and Difficulties Questionnaire total problems at age 11 according to child assessment compared to parent assessment (Inoue et al., 2021).

### Strengths

The challenges of establishing causal inference in studies of prenatal risk factors and childhood behavioral disorders are well described (Lewis et al., 2013). While our study faces many of these challenges, we used a unique data resource that captured assessments from various reporters at two different time points, allowing for a comparison of outcomes not only across reporters, but also over time, using the same sample and analytic methods. Furthermore, we used the school-aged ASEBA tools at both time points of follow-up further facilitating comparisons. An additional strength was the inclusion of the YSR during the adolescent follow-up, which provided a tool for self-assessment of behavior. We used analytic methods to account for non-participation.

### Limitations

Our findings should be interpreted in the context of limitations of the study design. Data on acetaminophen use were originally collected as part of a case-control study to investigate risk factors for a malformation, as such these data were reported retrospectively. While data were reported retrospectively, they were collected within a year of delivery on average, and thereby should not be influenced by childhood outcomes. Given the focus on early pregnancy exposures in the original study, medication use in late pregnancy was not explicitly asked about. Additionally, our study population included deliveries occurring from 1996–2002 and may not reflect current patterns of acetaminophen use. A final limitation regarding exposure is that we were unable to quantify dose in terms of milligrams of acetaminophen ingested per day as a large proportion of exposed study subjects were not able to recall dose. Consequently, we could not analyze how estimates changed across varying dose ranges. Our study is also susceptible to unmeasured confounding as we did not collect data on potential confounders such as parental IQ and parental mental health problems. Our study examined behavioral assessments conducted among a non-clinic sample using an assessment tool for subclinical behavior problems and therefore does not align with a specific diagnosis, such as ADHD or autism. While we report an attenuation of mean differences between exposed and unexposed groups over time, we were unable to assess how clinical diagnoses associated with prenatal acetaminophen exposure change over time. Lastly, dependent error, which arises when the same reporter is the source of both exposure and outcome data, may be driving observed associations with parent-reported outcomes (Kristensen, 1992).

The results of our study support that associations between prenatal exposure to acetaminophen and parent-reported behavioral outcomes wane over time from childhood to adolescence while teacher-reported behavioral outcomes were essentially null at both time points. While associations with parent- and teacher-reported outcomes were generally null at 11–17 years, there was a suggestion of a potential increase in behavioral problems according to youth self-report. More studies are needed to explore associations in later adolescence and to examine the effect of increasing age, potential time-varying confounders, genetic and epigenetic repercussions, dose range, and half-life of medications, which may impact associations.

## Data Availability

Data from this study are available upon request pending permission from the principal investigator and the Boston University Medical Campus Institutional Review Board. Requests to access the datasets should be directed to MW, werler@bu.edu.

## References

[B1] Achenbach T. M. McConaughy S. H. Howell C. T. (1987). Child/adolescent behavioral and emotional problems: Implications of cross-informant correlations for situational specificity. Psychol. Bull. 101, 213–232. 10.1037/0033-2909.101.2.213 3562706

[B2] Achenbach T. M. Rescorla L. A. (2001). Manual for the ASEBA school-age forms and profiles. Burlington, VT: University of Vermont, Research Center for Children Youth and Families.

[B3] Achenback T. Rescorla L. (2001). Manual for the ASEBA school-age forms and profiles. Burlington, VT: University of Vermont Research Centre for Children, Youth and Families, 80.

[B4] Andrade C. (2016). Use of acetaminophen (paracetamol) during pregnancy and the risk of autism spectrum disorder in the offspring. J. Clin. Psychiatry 77, e152–e154. 10.4088/JCP.16f10637 26930528

[B5] Bandoli G. Palmsten K. Chambers C. (2019). Acetaminophen use in pregnancy: Examining prevalence, timing, and indication of use in a prospective birth cohort. Paediatr. Perinat. Epidemiol. 34, 237–246. 10.1111/ppe.12595 31696962 PMC7192766

[B6] Bauer A. Z. Kriebel D. Herbert M. R. Bornehag C. G. Swan S. H. (2018). Prenatal paracetamol exposure and child neurodevelopment: A review. Hormones Behav. 101, 125–147. 10.1016/j.yhbeh.2018.01.003 29341895

[B7] Brandlistuen R. E. Ystrom E. Nulman I. Koren G. Nordeng H. (2013). Prenatal paracetamol exposure and child neurodevelopment: A sibling-controlled cohort study. Int. J. Epidemiol. 42, 1702–1713. 10.1093/ije/dyt183 24163279 PMC3887567

[B8] Damkier P. Pottegard A. dePont Christensen R. Hallas J. (2015). Annotations and reflections: Pregnancy and paracetamol: Methodological considerations on the study of associations between *in utero* exposure to drugs and childhood neurodevelopment. Basic and Clin. Pharmacol. Toxicol. 116, 2–5. 10.1111/bcpt.12322 25224918

[B9] Damkier P. (2020). Simple twist of fate: *In utero* exposure to acetaminophen and risk of childhood attention deficit hyperactivity disorder. Paediatr. Perinat. Epidemiol. 34 (3), 230–232. 10.1111/ppe.12654 32107788

[B10] Falt E. Wallby T. Sarkadi A. Salari R. Fabian H. (2018). Agreement between mothers', fathers', and teachers' ratings of behavioural and emotional problems in 3-5-year-old children. PLoS One 13, e0206752. 10.1371/journal.pone.0206752 30383861 PMC6211744

[B11] Golding J. Gregory S. Clark R. Ellis G. Iles-Caven Y. Northstone K. (2019). Associations between paracetamol (acetaminophen) intake between 18 and 32 weeks gestation and neurocognitive outcomes in the child: A longitudinal cohort study. Paediatr. Perinat. Epidemiol. 34, 257–266. 10.1111/ppe.12582 31523834 PMC7217049

[B12] Hartman C. A. Rhee S. H. Willcutt E. G. Pennington B. F. (2007). Modeling rater disagreement for ADHD: Are parents or teachers biased? J. Abnorm. child Psychol. 35, 536–542. 10.1007/s10802-007-9110-y 17333362

[B13] Inoue K. Ritz B. Ernst A. Tseng W. L. Yuan Y. Meng Q. (2021). Behavioral problems at age 11 Years after prenatal and postnatal exposure to acetaminophen: Parent-reported and self-reported outcomes. Am. J. Epidemiol. 190, 1009–1020. 10.1093/aje/kwaa257 33230558 PMC8248972

[B14] Kelley K. Kelley T. Kaufman D. Mitchell A. (2003). The slone Drug dictionary: A research driven pharmacoepidemiology tool. Pharmacoepidemiol Drug Saf. 12, S168–S9.

[B15] Kristensen P. (1992). Bias from nondifferential but dependent misclassification of exposure and outcome. Epidemiology 3, 210–215. 10.1097/00001648-199205000-00005 1591319

[B16] Lewis S. J. Relton C. Zammit S. Smith G. D. (2013). Approaches for strengthening causal inference regarding prenatal risk factors for childhood behavioural and psychiatric disorders. J. Child. Psychol. Psychiatry 54, 1095–1108. 10.1111/jcpp.12127 24007416

[B17] Liew Z. Bach C. C. Asarnow R. F. Ritz B. Olsen J. (2016). Paracetamol use during pregnancy and attention and executive function in offspring at age 5 years. Int. J. Epidemiol. 45, 2009–2017. 10.1093/ije/dyw296 28031314

[B18] Liew Z. Ritz B. Rebordosa C. Lee P. C. Olsen J. (2014). Acetaminophen use during pregnancy, behavioral problems, and hyperkinetic disorders. JAMA Pediatr. 168, 313–320. 10.1001/jamapediatrics.2013.4914 24566677

[B19] Liew Z. Ritz B. Virk J. Olsen J. (2015). Maternal use of acetaminophen during pregnancy and risk of autism spectrum disorders in childhood: A Danish national birth cohort study. Autism Res. 9, 951–958. 10.1002/aur.1591 26688372

[B20] Murray A. L. Booth T. Ribeaud D. Eisner M. (2018). Disagreeing about development: An analysis of parent-teacher agreement in ADHD symptom trajectories across the elementary school years. Int. J. Methods Psychiatr. Res. 27, e1723. 10.1002/mpr.1723 29845677 PMC6877228

[B21] Parker S. E. Collett B. R. Werler M. M. (2020). Maternal acetaminophen use during pregnancy and childhood behavioural problems: Discrepancies between mother- and teacher-reported outcomes. Paediatr. Perinat. Epidemiol. 34, 299–308. 10.1111/ppe.12601 31693212 PMC7192789

[B22] Rifas-Shiman S. L. Cardenas A. Hivert M. F. Tiemeier H. Bertoldi A. D. Oken E. (2020). Associations of prenatal or infant exposure to acetaminophen or ibuprofen with mid-childhood executive function and behaviour. Paediatr. Perinat. Epidemiol. 34, 287–298. 10.1111/ppe.12596 31637744 PMC7170759

[B23] Society for Maternal-Fetal Medicine Publications Committee (2017). Electronic address pso. Prenatal acetaminophen use and outcomes in children. Am. J. obstetrics Gynecol. 216, B14–B5.

[B24] Stergiakouli E. Thapar A. Davey Smith G. (2016). Association of acetaminophen use during pregnancy with behavioral problems in childhood: Evidence against confounding. JAMA Pediatr. 170, 964–970. 10.1001/jamapediatrics.2016.1775 27533796 PMC5300094

[B25] Stone S. L. Speltz M. L. Collett B. Werler M. M. (2013). Socioeconomic factors in relation to discrepancy in parent versus teacher ratings of child behavior. J. Psychopathol. Behav. Assess. 35, 314–320. 10.1007/s10862-013-9348-3 24043920 PMC3772786

[B26] Thompson J. M. Waldie K. E. Wall C. R. Murphy R. Mitchell E. A. (2014). Associations between acetaminophen use during pregnancy and ADHD symptoms measured at ages 7 and 11 years. PLoS One 9, e108210. 10.1371/journal.pone.0108210 25251831 PMC4177119

[B27] Tovo-Rodrigues L. Schneider B. C. Martins-Silva T. Del-Ponte B. Loret de Mola C. Schuler-Faccini L. (2018). Is intrauterine exposure to acetaminophen associated with emotional and hyperactivity problems during childhood? Findings from the 2004 pelotas birth cohort. BMC psychiatry 18, 368. 10.1186/s12888-018-1942-1 30458756 PMC6245767

[B28] Werler M. M. Sheehan J. E. Hayes C. Padwa B. L. Mitchell A. A. Mulliken J. B. (2004). Demographic and reproductive factors associated with hemifacial microsomia. Cleft Palate Craniofac J. 41, 494–500. 10.1597/03-110.1 15352870

[B29] Youngstrom E. Loeber R. Stouthamer-Loeber M. (2000). Patterns and correlates of agreement between parent, teacher, and male adolescent ratings of externalizing and internalizing problems. J. Consult Clin. Psychol. 68, 1038–1050. 10.1037//0022-006x.68.6.1038 11142538

[B30] Ystrom E. Gustavson K. Brandlistuen R. E. Knudsen G. P. Magnus P. Susser E. (2017). Prenatal exposure to acetaminophen and risk of ADHD. Pediatrics 140, e20163840. 10.1542/peds.2016-3840 29084830 PMC5654387

